# Mechanisms of nuclei growth in ultrasound bubble nucleation

**DOI:** 10.1016/j.ultsonch.2022.106091

**Published:** 2022-07-06

**Authors:** Matheus O. de Andrade, Reza Haqshenas, Ki Joo Pahk, Nader Saffari

**Affiliations:** a UCL Mechanical Engineering, University College London, London, United Kingdom; b Department of Biomedical Engineering, Kyung Hee University, Yongin, Republic of Korea

**Keywords:** Nucleation, Cavitation, Boiling, Histotripsy, CNT, Ultrasound, Bubble nuclei dynamics

## Abstract

This paper interrogates the intersections between bubble dynamics and classical nucleation theory (CNT) towards constructing a model that describes intermediary nucleation events between the extrema of cavitation and boiling. We employ Zeldovich’s hydrodynamic approach to obtain a description of bubble nuclei that grow simultaneously via hydrodynamic excitation by the acoustic field and vapour transport. By quantifying the relative dominance of both mechanisms, it is then possible to discern the extent to which viscosity, inertia, surface tension and vapour transport shape the growth of bubble nuclei through non-dimensional numbers that naturally arise within the theory.

The first non-dimensional number $\Phi_{1}^{2}/\Phi_{2}$ is analogous to the Laplace number, representing the balance between surface tension and inertial constraints to viscous effects. The second non-dimensional number δ represents how enthalpy transport into the bubble can reduce nucleation rates by cooling the surrounding liquid. This formulation adds to the current understanding of ultrasound bubble nucleation by accounting for bubble dynamics during nucleation, quantifying the physical distinctions between “boiling” and “cavitation” bubbles through non-dimensional parameters, and outlining the characteristic timescales of nucleation according to the growth mechanism of bubbles throughout the histotripsy temperature range.

We observed in our simulations that viscous effects control the process of ultrasound nucleation in water-like media throughout the 0–120 °C temperature range, although this dominance decreases with increasing temperatures. Enthalpy transport was found to reduce nucleation rates for increasing temperatures. This effect becomes significant at temperatures above 30 °C and favours the creation of fewer nuclei that are larger in size. Conversely, negligible enthalpy transport at lower temperatures can enable the nucleation of dense clusters of small nuclei, such as cavitation clouds. We find that nuclei growth as modelled by the Rayleigh-Plesset equation occurs over shorter timescales than as modelled by vapour-dominated growth. This suggests that the first stage of bubble nuclei growth is hydrodynamic, and vapour transport effects can only be observed over longer timescales. Finally, we propose that this framework can be used for comparison between different experiments in bubble nucleation, towards standardisation and dosimetry of protocols.

## Highlights

• We implement a CNT model that uses the Rayleigh-Plesset Equation to account for the growth of nuclei.
• Enthalpy transport creates cavitation clouds of small bubbles at low temperatures.
• Viscosity is the main constraint to nuclei growth in the 0–120 °C temperature range.
• The timescales of boiling bubble nucleation are higher than the timescales of cavitation.
• It is possible to distinguish the mechanism of nuclei growth via non-dimensional parameters.

## 1 Introduction

In previous papers, we have discussed how ultrasound nucleation pressure thresholds strongly depend on the medium's local temperature. Therefore, it is possible to use a temperature-dependent activity factor to harmonise theoretical CNT predictions and experimental data of ultrasound nucleation in the 1 – 2 MHz frequency range [1]. In this approximation, we assumed that nucleation takes place at the bottom most of an acoustic tensile wave, during a fraction of the acoustic period given as $\Delta t_{N}=\frac{1}{10f}$ that is sufficiently small to approximate nucleation rates as an isobaric, isothermal process. This model suggested that at room or physiological temperatures, the acoustic pressure significantly affects the rate at which bubbles nucleate. Conversely, nucleation rates increase up to 20 orders of magnitude between 60 and 100 °C, where the liquid's temperature is the driving parameter of the process [2], due to an increasing vapour pressure and a decreasing surface tension at high temperatures.

The theory derived in [1], [2] is a thermodynamic theory, which considers vapour transport as the sole mechanism involved in nuclei growth. Although that model can predict spatial–temporal trends of bubble nucleation within focused acoustic fields, it is skewed towards highlighting the energetic requirements of nucleation via the surface tension term in detriment of the effects of bubble radial dynamics. Therefore, the questions that this model cannot answer regard the common physical mechanisms of bubble growth between ultrasound cavitation [3], [4], [5], [6], [7] and boiling bubble nucleation [8], [9], [10], [11], [12], and how these mechanisms act as functions of temperature. This is because, in most practical applications, bubble growth is jointly determined by hydrodynamic oscillations caused by the acoustic pressure and thermal effects controlled by the liquid temperature such as vapour and gas transport [13].

A fundamental understanding of bubble nucleation is essential for the design of procedures that rely on the appearance and sustained activity of bubbles in a liquid medium, or to avoid the nucleation of bubbles when it is undesirable or hazardous. Focused ultrasound is an area where bubble nucleation is applied to create localised mechanical damage in soft tissue, via a technique named histotripsy. Histotripsy is a method where the ultrasound-induced nucleation and activity of bubbles inflicts mechanical injury to a focal volume whilst avoiding damage to overlying layers of tissue [14].

There are two broad categories of histotripsy which relate to the mechanism driving bubble nucleation. On the one hand, boiling histotripsy [15], [16] takes place at high temperatures in the presence of acoustic shockwaves of about −16 MPa peak negative pressure [1], [2]. On the other hand, cavitation histotripsy, usually classified as cavitation cloud [17], shock-scattering [18] or intrinsic threshold [7] histotripsy, occurs at physiological temperatures with peak-negative focal pressures around −30 MPa within as little as two ultrasound cycles. In-depth reviews of histotripsy and bubble dynamics can be found in [14], [19], [20].

Cavitation-based histotripsy techniques are remarkably repeatable when peak-negative pressure magnitudes surpass the liquids nucleation pressure threshold. In the intrinsic threshold method, a single bubble appears at the ultrasound focus [21], and the cavitating volume grows proportionally to the volume of the focal zone that surpasses the nucleation pressure threshold [22]. It has been observed that bubble maximum sizes are not strictly proportional to the magnitude of the peak-negative focal pressure, and that further increases in the magnitude of the incoming tensile wave will result in a greater number of bubbles nucleated within the focal zone [23]. After the growth stage of the bubbles is completed, they collapse inertially under ambient pressure [19], [23], unless residual internal gas content increases their longevity which is then controlled by passive diffusion [24], which caused histotripsy bubbles in tissue phantoms and a murine tumour model to be detectable via chirp-coded excitation up to 250 ms after sonication [25], [26].

Alternatively, boiling bubble nucleation usually happens following millisecond non-linear heat deposition due to the absorption of shockwaves [27]. The ultrasound focal volume transforms mechanical energy into heat until the temperature-dependent nucleation threshold equals the peak-negative ultrasound pressure at the focus, causing the appearance of a boiling bubble [2]. Numerical studies of boiling bubbles show that they can grow from nano to millimetre sizes by vapour and gas transport across their surface [9], [28], [29]. Their fully developed behaviour might also selectively induce mechanical damage to the parenchyma whilst sparing vascular tissue [30].

High-speed camera imaging of boiling bubble nucleation and growth in transparent tissue-mimicking phantoms shows that both processes of cavitation and boiling occur during boiling histotripsy [11]. In addition, there is evidence that the constructive interactions of the pressure waves reflected by boiling bubbles with the incoming acoustic field can create pre-focal regions of negative pressure that surpass the medium's nucleation pressure threshold, resulting in a cavitation cloud in the frontal side of the focus [31]. These tensile pressure regions have been observed in both linear and non-linear simulations of acoustic reflection from a vapour bubble [11], [31].

Herein, we analyse the role of thermal, inertial, surface tension and viscous effects in ultrasound-induced bubble nucleation. We employ a hydrodynamic formulation of classical nucleation theory, where the dynamics of spherical bubbles are considered by including the Rayleigh-Plesset [32] equation into the kinetic terms of a CNT model. This model is based on the hydrodynamic approach of Zeldovich developed in 1942 [33], further developed by Kagan and discussed in detail by Blander and Katz [34], [35], but, to the best of our knowledge, never investigated in the context of ultrasound nucleation in histotripsy.

## 2 Mathematical modelling

Our previous models of ultrasound nucleation [1], [2] were based on the Szilard model, where nucleation is thought to be a series of reactions between monomers (n=1 molecule bubble embryos) and polymers (n>1 molecule bubble embryos). This results in a steady-state nucleation rate that carries the assumption that bubble embryos can only grow from the evaporation of the surrounding liquid phase. In such a case, the nucleation rate is given by:

(1) $$J_{\mathrm{ss}}=\frac{\rho_{l}}{m}\sqrt{\frac{2\sigma }{\pi m}}\exp\left(-\Delta G^{*}\right),$$

where $\rho_{l}=\rho_{l}\left(T\right)$ [kg ∙ m^−3^] is the liquid density, and m [kg] the molecular mass. $\Delta G^{*}$ [J] is the free energy barrier for nucleation given in terms of the size of the critical nucleus as $\Delta G^{*}=\frac{W^{*}}{k_{B}T}=\frac{4}{3k_{B}T}\pi \sigma {r^{*}}^{2}$ [ND]. In this equation σ=σ(T) [J ∙ m^−2^] is the liquid's surface tension, $P_{v}$ and $P_{l}$[Pa] are, respectively, the vapour and acoustic pressure, $\zeta =1-\left(\frac{\rho_{v}}{\rho_{l}}\right)+\frac{1}{2}{\left(\frac{\rho_{v}}{\rho_{l}}\right)}^{2}$[ND], is a correction for nonideality defined in terms of the temperature-dependent density of water in the vapour $\rho_{v}$ and liquid $\rho_{l}$ phases. The radius of critical nuclei is then given by the Laplace equation of mechanical equilibrium $r^{*}=\frac{1}{\zeta }\frac{2\sigma }{P_{v}-P_{l}}$ [m], T [K] is the liquid's temperature, and $k_{B}$ [J ∙ K^−1^] is Boltzmann's constant.

The critical point of nucleation is a set of thermodynamic conditions in which the internal pressure of a nucleus exactly balances the pressure applied by the liquid and surface tension $P_{v}=P_{l}+\frac{2\sigma }{\zeta r^{*}}$. Therefore, any pair of pressure $P_{l}$ and temperature T such that $P_{l}=P_{l}^{N}\left(T\right)$ is a critical point. As discussed in detail in [1], [2], the temperature-dependent nucleation pressure threshold $P_{l}^{N}$ has the form:

(2) $$P_{l}^{N}=P_{v}-\frac{1}{\zeta }\sqrt{\frac{16\pi \sigma^{3}}{3k_{B}T\ln\left(\frac{J_{0}V_{0}\Delta t_{N}}{\Sigma }\right)}}.$$

In this equation, Σ is the number of critical nuclei formed in a volume $V_{0}$ during a time interval $\Delta t_{N}$ of the acoustic wave, and $J_{0}=\frac{\rho_{l}}{m}\sqrt{\frac{2\sigma }{\pi m}}$.

The factor $\frac{\rho_{l}}{m}$ in Eq. (1) accounts for the availability of monomers for bubble nucleation. This means that intrinsic thermal fluctuations of the system continuously create small nuclei which can trigger nucleation upon growing to a certain critical size $r^{*}$. From a thermodynamic point of view, these nuclei are transient, short-lived formations that happen due to the molecular movement in the liquid, which has a characteristic magnitude of $k_{B}T$. These events happen randomly in space and time, meaning that in any liquid, even at equilibrium, there are short-lived fluctuations in density that can be understood as bubble embryos. If one averages the number of fluctuations of all sizes that take place within a long observation window and divides this by the volume under investigation, the result is a spatially-averaged equilibrium distribution of nuclei $C\left(n,t\right)=C_{n}\left(t\right)$.

In a real nucleating system, nuclei will have an unknown distribution $Z\left(n,t\right)\equiv Z_{n}\left(t\right)$ that might be different to $C_{n}\left(t\right)$. The Szilard model establishes that the derivative $\frac{\partial Z_{n}}{\partial t}$ is simply the rate of arrivals at the size n subtracted by the rate of departures from size n. Let us denote $f\left(n,t\right)\equiv f_{n}$, as the rate at which n-sized embryos gain one vapour molecule, and $g\left(n,t\right)\equiv g_{n}$, as the rate that n-sized embryos lose one vapour molecule. This leads to the Master Equation of Nucleation [36], an expression of the form.

(3) $$\frac{dZ_{n}}{\mathrm{dt}}=f_{n-1}Z_{n-1}+g_{n+1}Z_{n+1}-\left(f_{n}+g_{n}\right)Z_{n}.$$

This equation means that the rate of arrivals at the size n will be given by the forward rate of n-1 sized nuclei growing into n added to the backward rate of n+1 sized nuclei shrinking into n. The rate of departures is given by n sized nuclei either growing or shrinking away from n. It would be difficult to establish the transition rates $f_{n}$ and $g_{n}$ individually, so we make use of a property of the equilibrium distribution. In equilibrium sizes are conserved [33], [36], therefore $f_{n}C_{n}=g_{n+1}C_{n+1}$ and $f_{n-1}C_{n-1}=g_{n}C_{n}$. By using thesse approximations for $g_{n}$, [27] shows that Eq. (3) assumes the differential form:

(4) $$\frac{\partial Z_{n}\left(t\right)}{\partial t}=\frac{\partial }{\partial n}\left\{f_{n}\left(t\right)C_{n}\left(t\right)\frac{\partial }{\partial n}\left[\frac{Z_{n}\left(t\right)}{C_{n}\left(t\right)}\right]\right\}=\frac{\partial }{\partial n}\left\{f_{n}\left(t\right)C_{n}\left(t\right)\left[\frac{1}{C_{n}\left(t\right)}\frac{\partial Z_{n}\left(t\right)}{\partial n}-Z_{n}\left(t\right){\left[\frac{1}{C_{n}\left(t\right)}\right]}^{2}\frac{\partial C_{n}\left(t\right)}{\partial n}\right]\right\}=-\frac{\partial }{\partial n}\left\{Z_{n}\left(t\right)v_{n}-f_{n}\left(t\right)\frac{\partial Z_{n}\left(t\right)}{\partial n}\right\}$$

This equation uses the equilibrium distribution of nuclei as a reference for calculating the true distribution of nuclei $Z_{n}\left(t\right)$ that might differ from $C_{n}\left(t\right)$, where the advective term in n is given by $v_{n}=f_{n}\left(t\right)\frac{\partial }{\partial n}\left[\ln C_{n}\left(t\right)\right]\equiv D_{n}\frac{\partial }{\partial n}\left[\ln C_{n}\left(t\right)\right]$. The full steady-state solution of Eq. (4) was discussed in detail by [36], resulting in an expression much like Eq. (1).

The influence of vapour pressures over nucleation decreases at lower temperatures and might become insignificant compared to very large tensile pressures at play in ultrasound nucleation [2]. Therefore, we implement a hydrodynamic model in order to accurately account for the growth of bubble nuclei in the low-temperature range. At these temperatures, bubble behaviour is highly sensitive to changes in the pressure field $P_{l}\left(t\right)$, but also limited by the liquid’s viscosity. This means that there is no one-to-one relationship between the number of vapour molecules inside a bubble and its radius. For example, a bubble with n internal vapour molecules will not have a unique radius r, but rather oscillate around an equilibrium value. In this paper, we bridge this gap by applying the approach of Zeldovich [33] to approximate the continuous size distribution $r_{n}⇄r_{n+1}$ in the radial coordinate r instead of the discrete transition n⇄n+1 in the size coordinate n. This will allow us to obtain the building blocks of a nucleation rate that accounts for how radial oscillations of a bubble nucleus affect its growth.

### 2.1 The generalised Zeldovich equation

We now wish to change the variables of Eq. (4) so that it uses the bubble radius as an independent variable. It is critical to notice that as a bubble embryo transitions from a size n to n+1, the differential in question is Δn=1 in the discrete coordinate system n. However, Δn is equivalent to an unknown number of radial variations dr in the continuous coordinate r. One can then assume that the transition from n to n+1, or from n to n-1 has a fundamental length scale $l_{r}=r\left(n+1\right)-r\left(n\right)$, where r(n) is a relationship derived from the ideal gas law. This length scale is useful in changing the independent variables of Equation (4) from n to r, resulting in.

(5) $$\frac{\partial Z\left(r,t\right)}{\partial t}=\frac{\partial }{\partial r}\left\{f_{r}l_{r}^{2}C_{r}\frac{\partial }{\partial r}\left[\frac{Z\left(r,t\right)}{C\left(r,t\right)}\right]\right\}=\frac{\partial }{\partial r}\left\{D_{r}C\left(r,t\right)\frac{\partial }{\partial r}\left[\frac{Z\left(r,t\right)}{C\left(r,t\right)}\right]\right\},$$

where $D_{r}\equiv f_{r}l_{r}^{2}$ has units of a diffusion coefficient. The Zeldovich model in Equation (4) employs $Z_{n}\left(t\right)$ as a probability mass function (PMF) of a discrete distribution of nuclei sizes. Differently to Eq. (4), Equation (5) employs Z(r,t) as a probability density function (PDF) of a continuous distribution of nuclei sizes. As a PDF, Z(r,t) has a central value $\overset{¯}{r}$ that changes as a function of time through the derivative $\frac{d\overset{¯}{r}}{\mathrm{dt}}$. This distribution is advected with velocity $v_{r}=f\left(\frac{\mathrm{dr}}{\mathrm{dt}},r,\cdots \right)$ and diffuses in the r coordinate with coefficient $D_{r}$.

It is now interesting to obtain the central value $\overset{¯}{r}$ of the hydrodynamic distribution Z(r,t), via the rate $\frac{d\overset{¯}{r}}{\mathrm{dt}}$. This will inform us of the mean radius of bubbles in the distribution, and whether the mean value is growing or shrinking as a function of time. We can approximate the expected value $\overset{¯}{r}$ as the centre of mass of Z(r,t) as:

(6) $$\overset{¯}{r}\left(t\right)=\underset{-\infty }{\int^{+\infty }}Z\left(r,t\right)rdr{\left(\underset{-\infty }{\int^{+\infty }}Z\left(r,t\right)dr\right)}^{-1}$$

where $\underset{-\infty }{\int^{+\infty }}Z\left(r,t\right)dr=N_{T}$ is the total number of nuclei at any t. We assume that this quantity is conserved if there are no bubble–bubble interactions. Zeldovich [33] has shown that the time-evolution of the mean size of nuclei in the distribution obeys the equation.

(7) $$\frac{d\overset{¯}{r}}{\mathrm{dt}}=\frac{1}{N_{T}}\int \frac{Z\left(r,t\right)}{C\left(r,t\right)}\frac{\partial }{\partial r}\left[D_{r}C\left(r,t\right)\right]dr\approx D_{r}\frac{d}{\mathrm{dr}}\left[\ln C\left(r,t\right)\right]$$

We can now develop Eq. (5) to obtain mathematical clarity on the role of the diffusive D and advective v terms in this equation:

(8) $$\frac{\partial Z\left(r,t\right)}{\partial t}=\frac{\partial }{\partial r}\left\{D_{r}C\left(r,t\right)\frac{\partial }{\partial r}\left[\frac{Z\left(r,t\right)}{C\left(r,t\right)}\right]\right\}=\frac{\partial }{\partial r}\left\{D_{r}C\left(r,t\right)\left[\frac{1}{C\left(r,t\right)}\frac{\partial Z\left(r,t\right)}{\partial r}-Z\left(r,t\right){\left[\frac{1}{C\left(r,t\right)}\right]}^{2}\frac{\partial C\left(r,t\right)}{\partial r}\right]\right\}=-\frac{\partial }{\partial r}\left\{Z\left(r,t\right)v_{r}-D_{r}\frac{\partial Z\left(r,t\right)}{\partial r}\right\}.$$

Equation (8) models the convection of $Z\left(r,t\right)$ with advective term $v_{r}\equiv \frac{d\overset{¯}{r}}{\mathrm{dt}}=D_{r}\frac{\partial }{\partial r}\left[\ln C\left(r,t\right)\right]$ and diffusion coefficient $D_{r}$.

**Fig. 1:**
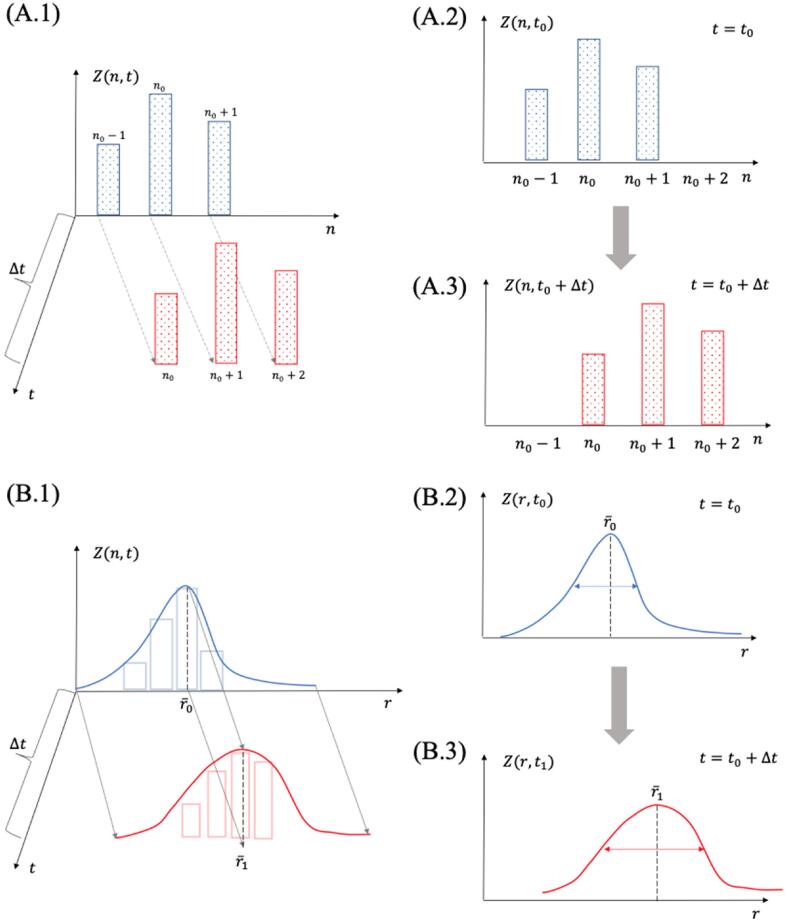
Schematic of the evolution of a nuclei distribution within the Szilard model (A.1, A.2, and A.3), where nuclei grow one molecule at a time and nuclei sizes are a discrete variable n compared to the evolution of a distribution within the Zeldovich model (B.1, B.2, and B.3) where nuclei grow hydrodynamically and nuclei sizes are a continuous variable r. The center of mass of the continuous distribution of nuclei is given by Eq. (6), and the width of the distribution is a measure of the diffusivity given by Eq. (10).

There are two factors at play in changing Z. The first, is that these embryos have a certain drift velocity $v_{r}$ that causes the average nucleus radius $\overset{¯}{r}$ to grow when $v_{r}>0$, to shrink when $v_{r}<0$, and to stay at the same size when $v_{r}=0$. This is to say that during nucleation, all nuclei in the distribution grow at a mean rate defined by $v_{r}$ and this drift velocity translates the concentration Z horizontally as shown in Fig. 1. The second important factor is that by subjecting the nuclei population to a pressure $P_{l}$ and a temperature T one might cause a diffusion in nucleus sizes with coefficient D. That is, the pressure field might make nuclei oscillate around their equilibrium radius. If the radius of nuclei oscillates about their equilibrium value, the width of the concentration Z(r,t) is constantly changed as the radius of every nucleus oscillates by ±dr.

Although centre of the distribution Z can be calculated via Eqs. (6), (7), Eqs. (4), (8) reveal how the spread of this distribution changes in time. Here, the unifying intersection between hydrodynamic and thermodynamic behaviour is given by D and v as.

(9.1) $$D_{n}=\frac{v_{n}}{\frac{\partial }{\partial n}\left[\ln C\left(n,t\right)\right]}=-\frac{k_{B}T\dot{n}}{\frac{\mathrm{dW}}{\mathrm{dn}}}=-\frac{k_{B}T\dot{n}\left(\frac{\mathrm{dn}}{\mathrm{dr}}\right)}{\frac{\mathrm{dW}}{\mathrm{dr}}}$$

(9.2) $$D_{r}=\frac{v_{r}}{\frac{\partial }{\partial r}\left[\ln C\left(r,t\right)\right]}=-\frac{k_{B}T\overset{˙}{\overset{¯}{r}}}{\frac{\mathrm{dW}}{\mathrm{dr}}}$$

The most important feature of these expressions is that there is no advection of the nuclei population at the critical point, where $\frac{\mathrm{dW}}{\mathrm{dr}}=\frac{\mathrm{dW}}{\mathrm{dn}}=0$, hence $\dot{n}=\dot{\overset{¯}{r}}=0$. Eqs. (9.1), (9.2) also tell us that the diffusion in nuclei size represented by D is proportional to the velocity at which the distribution travels the $\left(r,t\right)$ or (n,t) planes, and that size diffusion will be largest for bubbles with high wall velocities.

In both cases of vapour and hydrodynamic growth, Eqs. (9.1), (9.2) are a natural extension of the formula obtained by Einstein for the diffusion of a solute in a liquid solvent. Most importantly, there is a singularity in $D^{*}$. Since the variable r changes at much shorter timescales than n, it is of interest to check how radial changes affect the vapour flux. This is done by evaluating the critical diffusivity in Eq. (9.1) as a limit with respect to the critical radius by using L’Hôpital’s rule.

(10) $$D^{*}=\lim_{r\to r^{*}}D=\frac{k_{B}T{\left(\frac{d\dot{n}}{\mathrm{dr}}\right)}^{*}{\left(\frac{\mathrm{dn}}{\mathrm{dr}}\right)}^{*}}{{\left(\frac{d^{2}W}{dr^{2}}\right)}^{*}},$$

where $\left(\frac{\mathrm{dn}}{\mathrm{dr}}\right)$ is obtained from the ideal gas law.

#### 2.1.1 Nuclei growth by vapour and enthalpy transport

The rate of vapour transport $\dot{n}=\frac{\partial n}{\partial t}$ can be modelled in the presence of heat transfer from the liquid into the bubble as [34].

(11) $$\dot{n}=\frac{A\left(r\right)\left(P_{v}-P^{\prime }\right)}{\left(1+\delta \right)\sqrt{2\pi {\mathrm{mk}}_{B}T}}$$

where the non-dimensional factor (1+δ) [ND] accounts for a decrease in the influx $\dot{n}$ of molecules caused by enthalpy transport. Vapour transport into the bubble creates an enthalpy flux across the surface area A(r). The enthalpy of vapour is higher than that of liquid water, thus causing vapour to carry heat from the liquid into the bubble core. This transport decreases the temperature T around the bubble, and this change is captured by the non-dimensional quantity δ [34]:

(12) $$\delta =\sqrt{\frac{{2k}_{B}}{\pi mT}}{\left(\frac{\Delta H_{v}}{k_{B}T}\right)}^{2}\left(\frac{\sigma }{\lambda b}\right),$$

where λ is the thermal conductivity of the liquid [W ∙ m^−1^ ∙ K^−1^], $\Delta H_{v}$ [J] is the enthalpy of vaporisation of water, and $b=\frac{P^{\prime }-P_{l}}{P\prime }$ [ND] is the order of magnitude of the underpressure caused by the ultrasound wave in comparison to the nucleus internal pressure P′. The case where vapour carries insignificant heat through the bubble surface arises as the limit $\lim_{\delta \to 0}\dot{n}$.

#### 2.1.2 Nuclei growth by radial oscillations

To model the hydrodynamic growth of nuclei, Eq. (10) is evaluated with the Rayleigh-Plesset equation in conjunction with Eq. (11). The Rayleigh-Plesset equation establishes that the difference in pressure $\Delta P=P^{\prime }-P_{l}$ within and outside of a spherical bubble in an isothermal, incompressible liquid is given as [37]:

(13) $$P^{\prime }=P_{l}+\frac{2\sigma }{r}+\rho_{l}r\ddot{r}+\frac{3}{2}\rho_{l}{\dot{r}}^{2}+4\eta \frac{\dot{r}}{r},$$

where η=η(T) [Pa ∙ s] is the liquid's temperature-dependent viscosity, $\dot{r}$ is the bubble wall’s velocity and $\ddot{r}$ is its acceleration. Assuming that length scales of r are much smaller than the ultrasound wavelenght, we make the approximation $\frac{\partial P_{l}}{\partial r}=0$. We then employ the chain rule as $\ddot{r}=\frac{\partial \dot{r}}{\partial r}\frac{\partial r}{\partial t}$ to take the derivative of Eq. (13) with respect to r and evaluate it at the critical size, noticing that Eqs. 9 and 10 impose the boundary condition $\dot{r}=0$ when $r=r^{*}$. Therefore:

(14) $${\left(\frac{dP^{\prime }}{\mathrm{dr}}\right)}^{*}={\left\{-\frac{2\sigma }{r^{2}}+4\eta \left[\left[\frac{1}{r}\left(\frac{d\dot{r}}{\mathrm{dr}}\right)-\frac{\dot{r}}{r^{2}}\right]\right]+\rho_{L}\left[\left(\frac{d\dot{r}}{\mathrm{dr}}\dot{r}\right)+r\frac{d^{2}\dot{r}}{dr^{2}}\dot{r}+r{\left(\frac{d\dot{r}}{\mathrm{dr}}\right)}^{2}\right]+3\rho_{L}\dot{r}\left(\frac{d\dot{r}}{\mathrm{dr}}\right)\right\}}_{r^{*}}=-\frac{2\sigma }{{r^{*}}^{2}}+\rho_{l}r^{*}{\left[{\left(\frac{d\dot{r}}{\mathrm{dr}}\right)}^{*}\right]}^{2}+4\eta \frac{1}{r^{*}}{\left(\frac{d\dot{r}}{\mathrm{dr}}\right)}^{*}.$$

We are interested in solutions for all critical sizes $r^{*}$ that must also satisfy thermodynamic equilibrium [1], [2]. By restricting our solutions to those where ${P^{\prime }}^{*}\approx P_{v}$ we can make sure that size oscillations are purely driven by hydrodynamic forces, since this is a point of zero vapour flux [34]. By taking the derivative of $\dot{n}$ with respect to r at the critical size $r^{*}$ and removing vanishing terms:

(15) $${\left(\frac{d\dot{n}}{\mathrm{dr}}\right)}^{*}=\frac{A^{*}}{\left(1+\delta \right)\sqrt{2\pi {\mathrm{mk}}_{B}T}}\left\{\frac{2\sigma }{{r^{*}}^{2}}-4\eta \frac{1}{r^{*}}{\left(\frac{d\dot{r}}{\mathrm{dr}}\right)}^{*}-\rho_{L}r^{*}{\left[{\left(\frac{d\dot{r}}{\mathrm{dr}}\right)}^{*}\right]}^{2}\right\}.$$

This model can be closed by using an ideal gas law, such that $\dot{n}$ can be obtained in terms of the bubble radius and its derivatives in time [34]. The time derivative of an ideal gas law then takes the form.

(16) $$\dot{n}=\frac{\partial }{\partial t}\left(\frac{4}{3}\pi r^{3}\frac{P^{\prime }}{k_{B}T}\right).$$

The most significant limitations of an ideal gas law regard the effect of interactions between gas molecules, and the capacity to model liquid behaviour. Herein, the ideal gas law is used solely to model vapour phases, discarding the need for a thermodynamic account of the liquid phase. Moreover, an account of the effects of nonideality in gases is given by [35], where the authors find these to be negligible for the cases of boiling and condensation. By taking the derivative of Eq. (16) with respect to r, replacing Eq. (16) for $\frac{\partial P^{\prime }}{\partial r}$ then evaluating at the critical size yields the expression outlined by:

(17) $${\left(\frac{d\dot{n}}{\mathrm{dr}}\right)}^{*}=\frac{4\pi }{{3k}_{B}T}\left\{3P_{l}{r^{*}}^{2}{\left(\frac{d\dot{r}}{\mathrm{dr}}\right)}^{*}+4\sigma r^{*}{\left(\frac{d\dot{r}}{\mathrm{dr}}\right)}^{*}+4\eta {r^{*}}^{2}{\left[{\left(\frac{d\dot{r}}{\mathrm{dr}}\right)}^{*}\right]}^{2}+\rho_{l}{r^{*}}^{4}{\left[{\left(\frac{d\dot{r}}{\mathrm{dr}}\right)}^{*}\right]}^{3}\right\}.$$

#### 2.1.3 The effects of surface tension, inertia and viscosity

We can now equate Eqs. (17), (15), and define $\Gamma =\left(1+\delta \right)\sqrt{\frac{2\pi m}{k_{B}T}}$ [s ∙ m^−1^] and $X=\left(\frac{1}{4}\right)\Gamma r^{*}{\left(\frac{d\dot{r}}{\mathrm{dr}}\right)}^{*}$ [ND] as auxiliary variables. This yields a third-order hydrodynamic model that is written as a first-order non-linear differential equation of the third degree:

(18) $$\frac{\Phi_{2}}{\Phi_{1}^{2}}{\;X}^{3}+\left(\frac{8}{9}\right)\left(\frac{1}{\Phi_{1}}+\frac{27}{32}\frac{\Phi_{2}}{\Phi_{1}^{2}}\right)X^{2}+\left(\frac{2}{3}\right)\left(\frac{3-b}{b\Phi_{1}^{2}}+\frac{1}{\Phi_{1}}\right)X-\frac{1}{2\Phi_{1}^{2}}=0,$$

where the non-dimensional parameters $\Phi_{1}$ and $\Phi_{2}$ are defined as.

(19) $$\frac{\sigma T}{6\eta }=\frac{1}{\Phi_{1}},$$

and.

(20) $$\frac{16\rho_{l}r^{*}}{3\sigma \Gamma^{2}}=\Phi_{2}.$$

Upon analysis of Eqs. (19), (20), one will notice that $\Phi_{1}$ is a non-dimensional function of the Reynolds number $Re=\frac{\rho vL}{\eta }$, therefore, it is possible to characterise $\Phi_{1}$ as a metric of the effects of viscosity over inertia in nucleating bubbles. Similarly, $\Phi_{2}$ is a non-dimensional function of the Weber number $We=\frac{\rho v^{2}L}{\sigma }$, therefore it is possible to characterise $\Phi_{2}$ as a ratio between the fluid’s inertia and its surface tension. In the case where inertia is an important component, both Re and We assume large values and the process needs to account for inertial terms.

We can now make mathematical analogy with another fundamental non-dimensional number of fluid dynamics, the Laplace number $La=\frac{{\left(Re\right)}^{2}}{We}$, noticing that the ratio $\frac{\Phi_{2}}{\Phi_{1}^{2}}$ is a non-dimensional function of the Laplace number. In terms of the Reynolds, Weber and Laplace numbers, Eq. (18) becomes:

(21) $$La{\;X}^{3}+\left(\frac{8}{9}\right)\left(Re+\frac{27}{32}La\right)X^{2}+\left(\frac{2}{3}\right)\left(Re^{2}\frac{3-b}{b}+Re\right)X-\frac{1}{2}Re^{2}=0.$$

#### 2.1.4 Viscosity-dominated nucleation

The Laplace number is a measure of the surface and inertial forces as compared to the viscous forces in a bubbly flow. If the Laplace number is much greater than 1, it means that both surface tension and inertia dominate over viscous forces. Conversely, viscous forces dominate over both inertia and surface tension when La<1. Therefore, it is possible to approximate the limit where viscosity is the dominant parameter by assuming that La→0 and thus $\frac{\Phi_{2}}{\Phi_{1}^{2}}\to 0$ [34]. Eq. (21) then then is approximated by second-order polynomial where Y≈X at the limit of viscosity controlled nucleation $\frac{\Phi_{2}}{\Phi_{1}^{2}}\to 0$:

(22) $$Y^{2}+\left(\frac{3}{4\Phi_{1}}\right)\left(\frac{3-b}{b}+\Phi_{1}\right)Y-\frac{9}{16\Phi_{1}}=0.$$

### 2.2 The timescales of nucleation

We should note that cavitation-based histotripsy and boiling histotripsy take place at different timescales. Most notably, the intrinsic pressure threshold method for histotripsy is known to take place at very short insonation periods within two cycles of the acoustic wave [5], [7], [19], [21], [38] for ultrasound frequencies around 1 MHz. Conversely, boiling histotripsy takes place after several cycles (greater than1000) of the acoustic wave at the point where appreciable heat deposition has taken place [10], [14], [15], [39].

As noted by [40], after the ultrasound focal volume is brought to a metastable state, the system requires some time τ to achieve the steady-state nucleation rate $J_{\mathrm{ss}}$. The approach of a steady-state process is fundamentally dependent on the timescales required to establish a size distribution of nuclei Z(r,t) with values of r ranging from zero up to $r^{*}$ when starting with a pure fluid. In most practical cases, the time of occurrence of the first supercritical nucleus after the system has been brought to a metastable can be thought as the combination of three timescales: the nucleation time-lag τ, the time $\Delta t_{N}$ taken to form one critical nucleus for a steady-state nucleation rate $J_{\mathrm{ss}}$ in a volume $V_{0}$, and the time it takes for a nucleus to grow up to detectable dimensions $t_{G}$, for example, the relationships discussed in [41].

Originally, Zeldovich modelled τ as a Fourier-type number for the process of nucleation such that $\tau \propto \frac{{\left(r^{*}-r\right)}^{2}}{D^{*}}$ [33]. Similar approximations of the time-lag of nucleation have been obtained within one to two orders of magnitude of that obtained by Zeldovich. The determination of a precise form for τ depends on specific approximations that one makes when obtaining a transient solution to Eq. (8), as shown in [42], [43]. Herein, we employ the simpler approximation of Kashchiev [36] given by:

(23) $$\tau \approx \frac{10}{D^{*}}.$$

Calculating τ as a function of pairs of pressure and temperature $\left(P_{l}^{N},T\right)$ via $D^{*}\left(P_{l}^{N},T\right)$ will allow us to estimate the contributions of individual mechanisms in nucleation such as viscosity, inertia, vapour, and enthalpy transport to the overall timescales of the process.

### 2.3 Numerical methodology

**Fig. 2:**
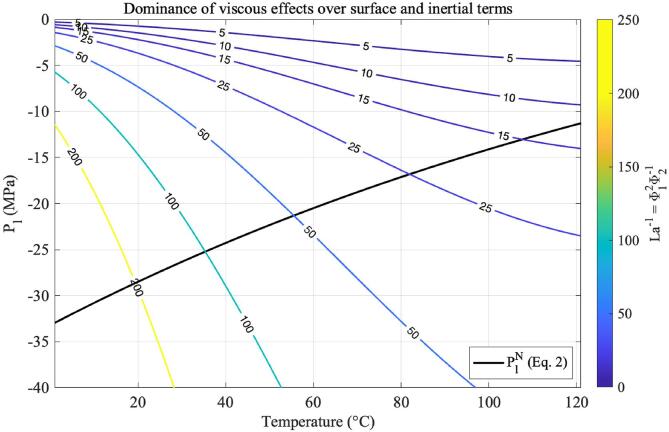
Values of the ratio $\Phi_{1}^{2}/\Phi_{2}$ across the histotripsy pressure and temperature range. Higher values of $\Phi_{1}^{2}/\Phi_{2}$ indicate that viscous effects dominate over the joint effects of surface tension and inertia.

**Fig. 3:**
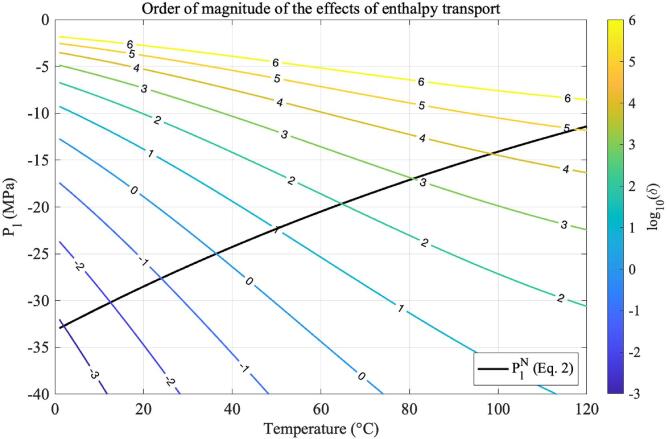
Values of $\log_{10}\delta$ across the histotripsy pressure and temperature range. Positive values of $\log_{10}\delta$ indicate that enthalpy transport at the bubble surface decreases the total number of bubbles nucleated because thermal energy is redirected onto making existing critical bubbles larger.

The results shown in Fig. 2, Fig. 3, Fig. 6, Fig. 7 are generated were generated for water in the temperature range 0 – 120 °C and pressure range −40 to 0 MPa, with an increment ΔT=1 [°C] and $\Delta P_{l}=5$ [kPa]. Since these quantities are not directly employed in solving differential equations, the resolution of the grid is set according to the desired numerical resolution. Furthermore, all results that are presented as critical are calculated at the pressure–temperature pairs $\left(P_{l}^{N},T\right)$, where $P_{l}^{N}$ is the temperature-dependent nucleation pressure threshold of water optimised for ultrasound nucleation as obtained in Eq. (2). The fact that $P_{l}^{N}$ depends on temperature means that although both pressure and temperature are thermodynamic variables of an experiment, the model has the liquid temperature as the sole degree of freedom.

The International Association for the Properties of Water and Steam (IAPWS) formulation was employed to calculate water's physical properties. Physical constants and expressions for thermodynamic properties of water were taken from the 1995 release by the IAPWS [44] and related subsequent releases. The free energy of nucleation and nucleation pressure thresholds $P_{l}^{N}$ were calculated with the modified surface for ultrasound-induced nucleation discussed in detail in [1], [2]. We have assumed an average timescale of $\Delta t_{N}=\frac{1}{2\times 10^{7}}$ for nucleation to take place at the bottom of the peak-negative pressure of a 2 MHz histotripsy shockwave. This was shown to be an average time that produces small error in $P_{l}^{N}$ in the frequency range 1 – 2 MHz, as discussed in [2].

We would like to bring the attention of the reader that in [1], [2] we discuss how the agreement between Eq. (2) and experimental results as listed in Table 3 is subject to approximating σ with an effective value $\sigma_{E}$ that is a function of temperature in the free energy term $\Delta G^{*}$. This approximation was based on the approach of [5], [45] when analysing bubble nucleation data. One important subtlety in the work of [46] suggests that this type of approximation is, in theory, not restricted to the surface tension itself, but rather an approximation made for the free energy barrier $\Delta G^{*}$. Differently from [5], [45], the work in [46] did not intend to harmonise theoretical and experimental results, but rather formulate a description of $\Delta G^{*}$ that vanishes at the spinodal limit of water. Therefore, the calculations shown in this paper assume that σ is the scaled surface tension $\sigma_{E}=\Psi_{E}\sigma \;$ obtained in [2] and used to calculate $P_{l}^{N}$ according to Eq. (2) throughout this work.

By solving Eq. (2) to obtain the temperature-dependent nucleation pressure threshold, one can calculate the value of $r^{*}$ as discussed in [1], [2]. These results are used to calculate critical values of $\delta ,\Phi_{1}$ and $\Phi_{2}$ as described by Eqs. (12), 19 and 20, enabling the evaluation of $D^{*}$ via Eq. (10), and of τ via Eq. 24. In sum, Eq. (18) can be solved numerically to obtain values of X for the third-order approximation, that includes the effects of inertial forces. Likewise, Eq. (22) can be solved numerically to obtain values of the second-order approximation Y. The solution of Eqs. (18), (22) will yield multiple results, and only the largest real-valued solutions are considered for analysis.

In addition to the numerical analysis described above, qualitative analysis of results was performed by comparing the model's predictions developed herein with experimental results in the literature as presented in Table 1, Table 2, Table 3.

**Table 1:** Boiling histotripsy experimental parameters at around 100 °C.

**Reference**	f(MHz)	**Tissue**	$p^{+}$(MPa)	$p^{-}$(MPa)
[27]	2.158	Ex vivo bovine liver	36	9
		Ex vivo bovine heart	70	12
[47]	2.158	Ex vivo bovine liver	67	12
[48]	2.158	Ex vivo bovine heart	67	12
		Tissue-mimicking gel	76	13.5
[49]	2.158	Ex vivo bovine heart	73	12
		Ex vivo bovine liver	73	12
[50]	1.1	Ex vivo porcine liver In vivo rat liver	74	14.4
	2.0		101	16.7
[51]	2.0	In vivo rat liver	94	15
			101	17
[11]	2.0	Tissue-mimicking gel	85.4	15.6
[52]	1.5	In vivo carcinoma Eker rat	85	17
			100	20
[53]	1.5	In vivo porcine kidney and liver	80	12–18
[54]	2.0	Human breast adenocarcinoma	85	14
[8]	2	Tissue-mimicking gel	89.1	14.6
	3.5		72.4	13.8
	5		69.2	12.5

**Table 2:** Cavitation histotripsy experimental parameters at room temperature.

**Reference**	f(MHz)	**Tissue**		$p^{-}$(MPa)
[55]	0.75	Ex vivo porcine heart		22
[56]		Ex vivo rabbit kidney		22
[57]		Ex vivo canine prostate		20
[58]	1	Tissue-mimicking gel		19
[21]	1.1	Distilled water 10% O_2_		27.4
		Unfiltered water 90% O_2_		26.2
		Tissue-mimicking gel (5%)		27.3
		- (15%)		28
		Ex vivo canine blood		26.9
		Ex vivo canine blood clot		26.8
		Ex vivo canine kidney		29.4
[59]	1	In vivo porcine liver		17
[60]	0.5	Ex vivo canine kidney		28.5
		Ex vivo canine liver		29.3
		Tissue-mimicking gel		24.5
[61]	1	Blood clot		36

**Table 3:** Temperature-dependent nucleation thresholds in water.

Reference	f(MHz)	$p^{-}$(MPa)	Mean error (MPa)	T(°C)
[3], [5]	1.1	34	±1	1
		33		3
		34		5
		32		7
		32		9
		31		11
		31		13
		30		16
		30		20
		28		29
		26		38
		25		48
[7]	1	29.8	0.7	10
		28.9	0.6	20
		24.7	1.9	40
		21.8	2	60
		17.4	2.4	80
		14.9	3.5	90

## 3 Results

### 3.1 The relative importance of viscous, surface tension and inertial effects in ultrasound bubble nucleation

The extent where viscous effects dominate over surface tension and inertial effects in ultrasound nucleation can be visualised as a function of pressure and temperature in Fig. 2. In this figure, the black solid curve represents the ultrasound temperature-dependent nucleation pressure threshold as calculated from Eq. (2), and the coloured contours illustrate values of the ratio $\Phi_{1}^{2}/\Phi_{2}$. The quantity $\Phi_{1}^{2}/\Phi_{2}$ is analogous to the inverse of the Laplace number, where $\frac{\Phi_{1}^{2}}{\Phi_{2}}\propto La^{-1}=\frac{27}{4}\frac{\eta^{2}}{\sigma \rho_{l}r^{*}}$. At values of $\frac{\Phi_{1}^{2}}{\Phi_{2}}>1$, one can say that viscosity dominates over the joint effects of surface tension and inertia in bubble nucleation.

Fig. 2 shows that the dominance of viscosity is particularly visible at low temperatures, indicating that nucleation in cavitation-based histotripsy methods is controlled by the liquid viscosity to a greater extent than the hydrodynamic effects of surface tension and inertia at the bubble surface. In particular, the ratio $\Phi_{1}^{2}/\Phi_{2}$ stays within the range 200 – 100 for normothermic temperatures (20 to 40 °C) and around histotripsy intrinsic threshold pressures (-40 to −25 MPa). At higher temperatures, viscous effects are less pronounced, and the ratio $\Phi_{1}^{2}/\Phi_{2}$ stays within the range of 25 – 10 around pressure–temperature pairs compatible with boiling histotripsy bubble nucleation, from 80 to 120 °C and from −5 to −25 MPa.

Similarly, the order of magnitude of the effects of heat transport given by $\log_{10}\delta$ is shown as a function of pressure and temperature in Fig. 3. In this figure, the black curve represents the ultrasound temperature-dependent nucleation pressure threshold as calculated by Eq. (2), and the coloured contours illustrate values of the quantity $\log_{10}\delta$. Positive values of $\log_{10}\delta$ will indicate extensive influence of enthalpy transport across the bubble surface in the nucleation process. As proposed by [28], enthalpy transport across the nucleus surface will cool down the surrounding liquid. This effect causes the liquid to lose supersaturation in the vicinity of critical bubbles via an increase in the energy barrier to nucleation $\Delta G^{*}$. This effect will favour the growth of the first bubbles to nucleate in detriment of a decrease in the number of bubbles nucleated subsequently.

According to the results shown in Fig. 3, such heat transport effects are more pronounced at temperatures above 40 °C. In particular, the order of magnitude of δ increases by a factor of three in the temperature range 60–100 °C. Conversely, at temperatures below 40 °C, the effects of heat transport seem to be negligible, and the order of magnitude of δ ranges from $10^{-2}$ to $10^{0}$. The immediate physical implication of this analysis is that nucleation at low temperatures, like cavitation-based histotripsy, occurs in a regime where the nucleation of the first few nuclei does not hinder the nucleation of subsequent nuclei. This is an environment where it is thermodynamically favourable for nucleation to occur in densely populated clouds of small bubbles. Conversely, nucleation favours smaller quantities of bubbles of larger size at high temperatures.

### 3.2 The effects of enthalpy transport

**Fig. 4:**
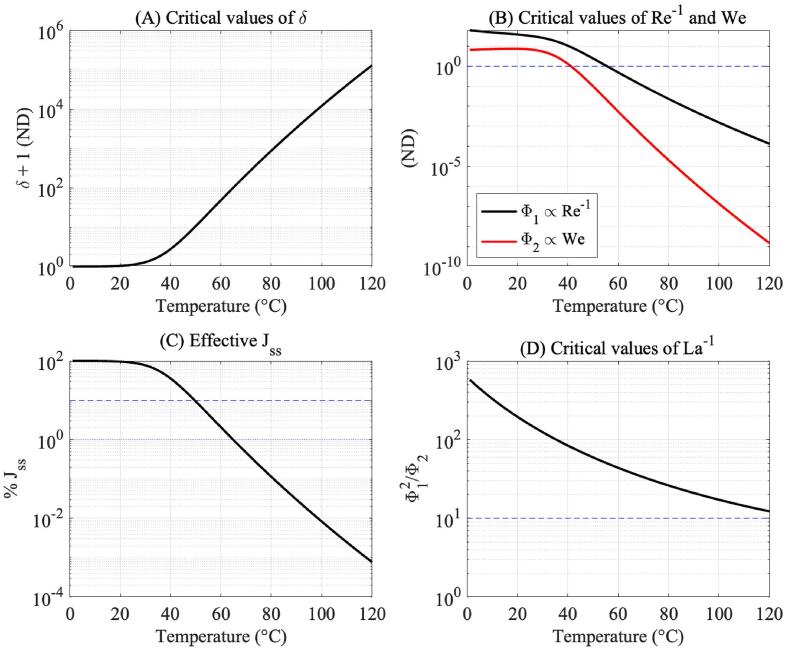
(A) Values of the term $\left(\delta +1\right)$ representing the enthalpy flux across the bubble surface over the ($P_{l}^{N},T^{N}$) nucleation curve, (B) values of $\Phi_{1}$ and $\Phi_{2}$ over ($P_{l}^{N},T^{N}$) (C) percent estimate of the effective value of $J_{\mathrm{ss}}$ due to the effect of enthalpy transport, and (D) values of the ratio $\Phi_{1}^{2}/\Phi_{2}$ over the ($P_{l}^{N},T^{N}$) curve.

Figure 4-A illustrates critical values of the constant (δ+1) as a function of temperature. These are the values of $\delta^{*}=\delta \left(P_{l}^{N},T^{N}\right)$ calculated at pressure–temperature pairs obtained with Eq. (2). This non-dimensional term appears in the definition of $\dot{n}$ because of the effects of heat and vapour transport into the bubble nucleus. The enthalpy of vapour is higher than that of liquid water, and as water changes from liquid to a vapour phase, it absorbs thermal energy from the surroundings of the bubble nucleus [34]. It can be observed in Fig. 4-A that this effect increases with increasing temperature, which results in a decrease in the nucleation rate of vapour bubbles shown in Fig. 4-C. As numerical examples, the ratio $\frac{1}{1+\delta }$ takes on values of 0.9664 at 20 °C, 0.3996 at 40 °C, 0.0240 at 60 °C, and 9.4466 × 10^−5^ at 100 °C. Fig. 4-C then shows that this effect reduces nucleation rates by 60% at 40 °C and by over 99% around 100 °C. These results again suggest that dense cavitation clouds appearing at low temperatures are a consequence of negligible vapour transport into the nuclei population characterised by small values of δ. This is a case in which the nucleation of bubbles does not change the free energy $\Delta G^{*}$ available for new bubbles to nucleate.

Moreover, critical values of the constants $\Phi_{1}$ and $\Phi_{2}$ are plotted as functions of temperature in Fig. 4-B. These non-dimensional terms originate from the non-linear ordinary differential equations in Eqs. (18), (22), which describe hydrodynamic effects in bubble nucleation. If analysed in terms of the Reynolds number Re and the Weber number We, these results indicate that viscous forces dominate over inertial forces at low temperature, as shown by $\Phi_{1}\propto Re^{-1}$, however $\Phi_{2}\propto We$ indicates that inertial effects overshadow surface tension effects at lower temperature. It is important to highlight that the definition of both $\Phi_{1}$ and $\Phi_{2}$ is inversely proportional to δ, and the decrease of these quantities at high temperatures is likely to be linked to an increase in the extent of enthalpy transport effects via high values of δ as shown in Fig. 4-A. More information can be drawn from these results by then analysing Fig. 4-D. These results show that the dominance of viscosity over both surface tension and inertia reduces with increasing temperature.

### 3.3 The mechanisms and timescales of nuclei growth

**Fig. 5:**
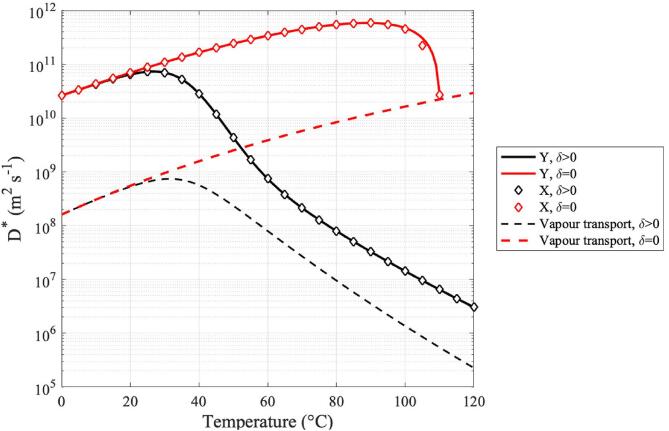
Critical diffusivity coefficients for vapour transport, heat transport and hydrodynamic growth. Red curves represent nuclei growth in the absence of enthalpy transport, whilst black curves represent growth in the presence of enthalpy transport. The largest real-valued positive solutions for X and Y from Eqs. (18), (22), respectively, are selected as representing solutions for the third and second-order hydrodynamic approaches.

In Fig. 5, the critical diffusivity defined in Eq. (10) is calculated along $\left(P_{L}^{N},T^{N}\right)$ for the mechanisms of vapour and hydrodynamic growth with and without the presence of enthalpy transport. In this figure, curves shown in black represent values of X, Y and $\dot{n}$ as given by Eqs. (22), 18, and 11 for the case where enthalpy transport is present, characterised by δ>0. Conversely, curves shown in red represent the cases where enthalpy transport is neglected, which is characterised by δ=0. Eq. (18) is a third order polynomial, therefore it has at least one real root of X, whereas the other two roots might be either a pair of real roots or a pair of complex conjugate roots. Moreover, Eq. (22) has one pair of real roots, where one is positive, and the other is negative. In this figure, we show results for the largest real-valued positive roots of X and Y. In accordance to Fig. 4-D, where $\frac{\Phi_{1}^{2}}{\Phi_{2}}\gg 1$ throughout the temperature range of interest, we can observe that there is no appreciable distinction between hydrodynamic growth dominated by viscosity and that dominated by inertial effects.

**Fig. 6:**
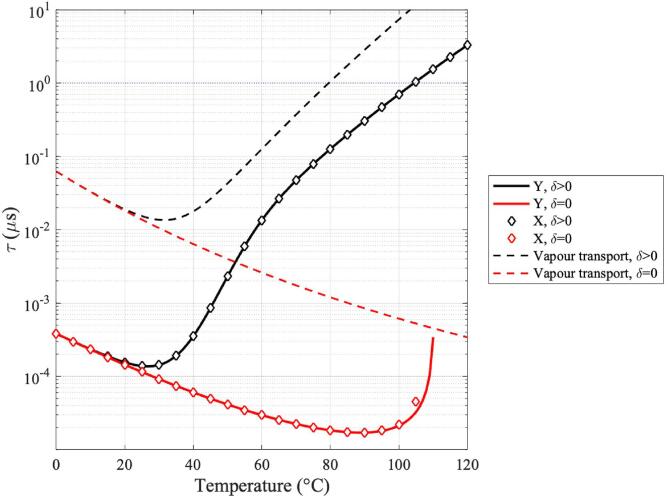
Nucleation time-lag for bubble nucleation as controlled by vapour transport, heat transport and hydrodynamic growth. Red curves represent nuclei growth in the absence of enthalpy transport, whilst black curves represent growth in the presence of enthalpy transport.

**Fig. 7:**
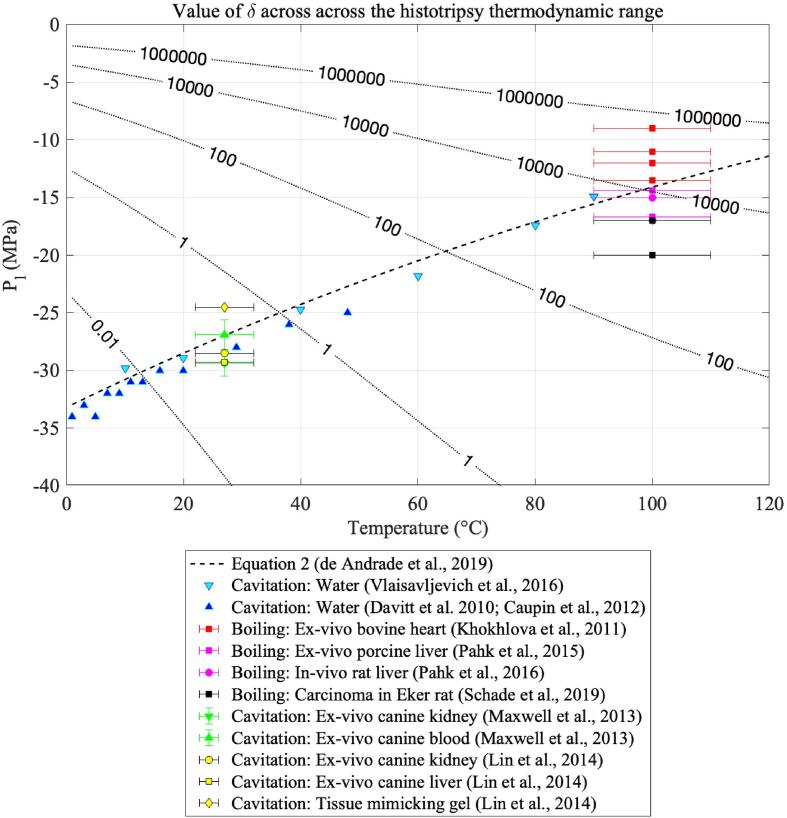
Values of δ across the histotripsy range of pressures and temperatures.

**Fig. 8:**
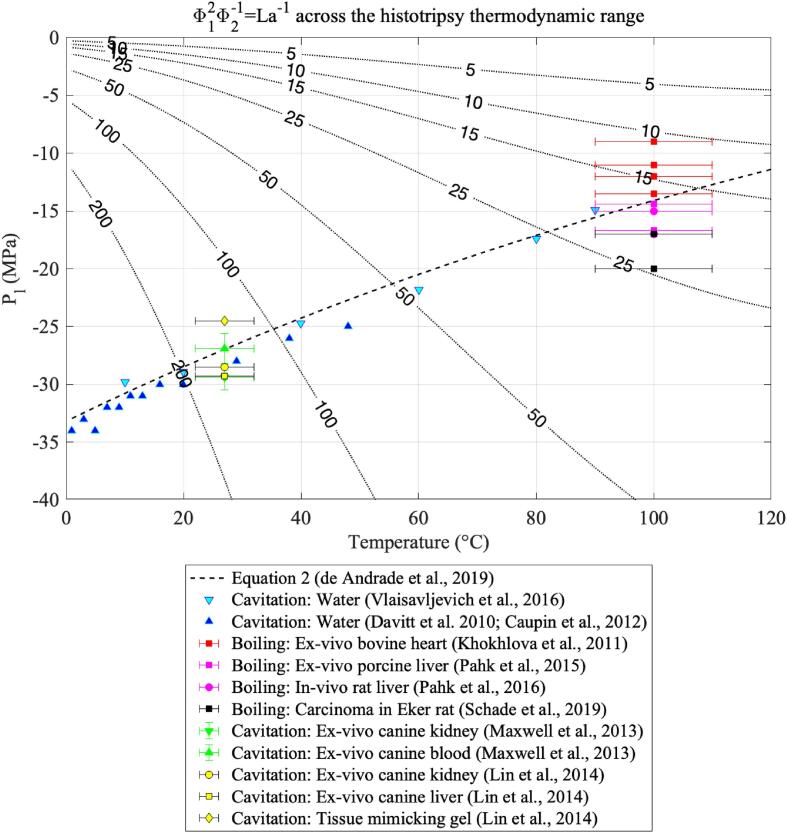
Values of $\Phi_{1}^{2}/\Phi_{2}$ across the histotripsy range of pressures and temperatures.

These results show that hydrodynamic nuclei growth rates are at least one order of magnitude greater than growth caused solely by vapour transport throughout the temperature range of interest. Moreover, the results illustrated in Fig. 5 show that the mechanism of enthalpy transport greatly reduces nuclei growth as given by the critical diffusivity in Eq. (10). The critical diffusivity coefficients present a turning point around 30 °C, which if analysed in conjunction with the inflection point in Fig. 4-A, might be interpreted as the point where enthalpy transport starts to play a role in nucleation. We can observe close agreement between the third and second-order hydrodynamic approximations throughout the 0 – 120 °C temperature range, regardless of the presence of enthalpy transport. This can be explained by the results shown in Fig. 4-D, showing that the Laplace number $\frac{\Phi_{1}^{2}}{\Phi_{2}}\gg 1$ for the temperature range considered, and thus viscous effects dominate the process.

Fig. 6 shows the nucleation time-lag as calculated via Eq. (23) for each of the mechanisms of nuclei growth considered herein. The magnitude of the time-lag of nucleation gives us an indication of the mechanism that allows the system to achieve steady-state nucleation rates in a shorter time. These results show that the timescales of nucleation as induced by hydrodynamic factors are up to two orders of magnitude faster than those characteristic to nucleation caused solely by vapour transport. These results suggest that the growth of nuclei is immediately controlled by hydrodynamic effects caused by the presence of an acoustic field.

As discussed in [40], τ is a fundamental timescale of nucleation, which reflects the contributions of the mechanism of nuclei growth to the time-dependency of the process. The approximation in Eq. (23) is an upper limit to the values of τ, under the common assumption that only approximately 10% of critical nuclei can become supercritical and grow onto fully developed bubbles. The interpretation of [62] is that τ is a measure of the meantime that it takes for a nucleus to undergo a critical growth $\Delta^{*}r$ such that nucleation occurs. Therefore, this quantity is extremely sensitive to the underlying mechanism of nucleus growth given by $D^{*}$.

More interestingly, Fig. 6 shows that the time-lag of nucleation increases with increasing temperature. Although the nucleation time-lag is of the order of nanoseconds at temperatures below 50 °C, these timescales increase up to microsecond scales at around 100 °C. Numerical examples of the ratio between the nucleation time-lag and the experiment time $\Delta t_{N}$ used to calculate nucleation pressure thresholds in Eq. (2) for hydrodynamic nucleation are $\frac{\tau }{\Delta t_{N}}=8\times 10^{-4}$ at 20 °C, $7\times 10^{-4}$ at 30 °C, $1.5\times 10^{-3}$ at 40 °C, $5.78\times 10^{-2}$ at 60 °C, $5.73\times 10^{-1}$ at 80 °C and 3.23 at 100 °C. These results show not only increasing timescales with temperature, but also suggest that a transient treatment of nucleation at temperatures above 80 °C would yield new information into the process of bubble formation at high temperatures.

### 3.4 Classifying and comparing different ultrasound nucleation results

In Fig. 6, the constant δ is compared to histotripsy experimental protocols in the literature. It can be observed that all normothermic intrinsic pressure threshold histotripsy protocols take place at pressure–temperature pairs where δ ranges from $10^{-2}$ to $10^{0}$. On the other hand, most boiling histotripsy protocols are performed at pressure–temperature pairs where δ ranges from $10^{2}$ to $10^{5}$. This is evidence that there is considerable vapour and heat transfer into the bubble in boiling histotripsy protocols, which, as exemplified by Fig. 4-C, causes a decrease in the net number of bubbles nucleated.

This result agrees with high-speed imaging of boiling histotripsy protocols, where boiling bubbles appear in greater size but smaller quantities at the distal side of the focal region [11]. On the other hand, small values of δ at intrinsic histotripsy pressure–temperature pairs suggests that no significant heat transport takes place into the nuclei, and these are free to nucleate in higher quantities. Again, this correlates with documented experimental results, which report the appearance of densely populated clouds of bubbles for intrinsic threshold histotripsy [11], [63], [64].

Finally, Fig. 7 shows values of the ratio $\Phi_{1}^{2}/\Phi_{2}$ as compared to histotripsy protocols. These results show $\Phi_{1}^{2}/\Phi_{2}$ assumes values within the range 200 – 100 when bubble nucleation occurs within the pressure range from −40 to −20 MPa, indicating that the liquid's viscosity plays a significant effect in nucleation at these pressure–temperature pairs. Conversely, when bubble nucleation takes place within the pressures ranging from −20 to 0 MPa and temperatures within 80 to 120 °C, $\Phi_{1}^{2}/\Phi_{2}$ assumes values between 25 and 10, indicating that the liquid's viscosity has a less pronounced effect in bubble nucleation at higher temperatures. Concerning histotripsy, these results indicate that the liquid's viscosity plays a significant effect in defining nucleation pressure thresholds for cavitation-based histotripsy approaches, and this effect is less critical for boiling histotripsy.

### 3.5 Overview of the model, limitations, and directions for future work

In this paper, we have applied the hydrodynamic theory of nucleation developed by Zeldovich and furthered by Kagan and Blander into analysing the role of thermal and hydrodynamic constraints on the growth of bubbles in ultrasound nucleation using histotripsy as case-study. The Zeldovich theory is one that circumvents the need for having a priori information on the initial distribution of bubbles by analysing how the size distribution of nuclei Z(r,t) evolves in time as compared to the equilibrium distribution of nuclei C(r,t) from liquid kinetics. This is a convenient mathematical framework since it is applicable to distributions of all sizes and shapes if one can establish a relationship between the nuclei population under consideration to an equilibrium population of nuclei.

The present work furthers our understanding in ultrasound bubble nucleation by relating the direct effects of ultrasound pressure fields characterised by the drift v to near-equilibrium effects characterised by the critical diffusivity D in terms of the liquid’s temperature. This allows us to construct a fundamental set of equations which yields non-dimensional measures of the relative effects of constraints such as viscosity, inertia, surface tension and enthalpy transport in bubble nucleation. When compared to documented experimental data in bubble nucleation and histotripsy, these metrics outline well-defined parameter windows where nucleation takes place via equivalent mechanisms. The immediate implication of these results is that metrics such as Eqs. (12), 19 and 20 can be used to compare the equivalence and similarity of protocols for ultrasound bubble nucleation in water.

It is important to outline that amongst all constraints analysed for bubble nucleation, the surface tension of bubble nuclei is the only one that is present in the two fundamental components of nucleation, acting both as a kinetic term and an energetic term. The surface tension acts as an energetic term because it is very closely related to the energy barrier that needs to be overcome such that nucleation takes place, given by $\Delta G^{*}$ in Eq. (2). Alternatively, the surface tension affects the kinetics of bubble nucleation because it is an active term in determining both the radial dynamics of bubbles as given by the Rayleigh-Plesset equation in Eq. (13), and the extent to which enthalpy transport decreases vaporisation rates into bubble nuclei via Eq. (12). Therefore, it is important to highlight that, although the viscosity of the surrounding liquid is the dominant factor with respect to the growth of bubble nuclei, the surface tension remains the most critical parameter in nucleation, because the nucleation rate depends on it exponentially as shown in Eq. (1).

Finally, we hope to clarify to the reader that the present model is based on several models present in the literature, many times developed as local approximations. For example, as discussed in [1], [2], our thermodynamic model of bubble nucleation assumes an isobaric and isothermal liquid, so that the work of nucleation can be constructed via the Gibbs free energy potential. For this assumption to be valid, the nucleation pressure threshold described by Eq. (2) is obtained within one tenth of the acoustic cycle (approximately 50 ns), such that the pressures and temperatures in the surrounding liquid can be considered constant. Furthermore, the derivation of the nucleation time-lag assumes that although the true distribution of nucleus sizes Z(r,t) might be different to C(r,t), their ratio is somehow constant as outlined in [43], where the definition of τ is, in fact, a statement of the domain where this assumption is valid.

Future work might build up on these results by investigating transient nucleation in ultrasound in the context of the models given by [40], [42], [43], analysing the time-evolution of nucleation with respect to the ultrasound waveform $P_{l}\left(t\right)$. Since the definition of Eqs. (4), (8) allow for general nuclei distributions, this model might also be applicable to investigate acoustic propagation in bubbly flows where there is a direct relationship between the bubble population and the local pressure field. Moreover, an interesting possibility for validation of our results would be to employ models of bubble dynamics in ultrasound pressure fields [29], [39] for a large window of temperature-dependent parameters to investigate whether there are visible trends of bubble dynamics associated with the two main regimens of nuclei growth discussed herein.

## 4 Conclusions

A hydrodynamic model for ultrasound-induced bubble nucleation was obtained by including the effects of the liquid's viscosity and inertia via the Rayleigh-Plesset equation in a classical nucleation theory model. In addition, the effects of heat transport into the bubble were accounted for by including a model of enthalpy transport across the bubble surface. This approach was instrumental in calculating the critical diffusivity of nucleation, which affects the rate at which bubbles nucleate and grow in ultrasound pressure and temperature fields.

With the hydrodynamic approach considered herein, it is possible to classify bubble nucleation concerning its dominant mechanism. Bubble nucleation at temperatures below 30 °C is shown to be largely dependent on the liquid's viscosity, with negligible influence of inertial effects or heat transport. On the other hand, bubble nucleation at high temperatures has a much weaker dependence on viscous constraints in the liquid and is mainly controlled by heat transport into the bubble.

The timescales of nucleation as outlined by this model are in qualitative agreement with those of boiling and cavitation histotripsy reported in the literature. The fundamental timescale of nuclei growth, namely the nucleation time-lag τ, increases with increasing temperature and is of the order of one microsecond at boiling temperatures (100 °C) and of the order of nanoseconds at room temperatures, in agreement with our previous modelling of the histotripsy process. We find that the timescales for hydrodynamic growth, as imposed by the radial oscillations of critical nuclei, are at least two orders of magnitude smaller than those that are characteristic of vaporisation-dominated growth. This means that bubble nuclei first grow via hydrodynamic factors, which are supplemented by vapour transport over longer timescales.

Notably, the enthalpy transport effect is a feasible explanation of the mechanism involved in the formation of cavitation clouds in ultrasound-induced nucleation. At higher temperatures, vapour flux into the bubble reduces the temperature of its surroundings because the enthalpy of vapour is greater than the enthalpy of liquid water. This cooling effect decreases the supersaturation around boiling bubbles, which then appear in greater size and smaller quantities. Conversely, negligible enthalpy transport at temperatures below 30 °C leads to higher nucleation rates of smaller bubbles, resulting in the nucleation of clusters of small gas pockets, where a local nucleation event does not affect the likelihood of nucleation in its surroundings.

## CRediT authorship contribution statement

**Matheus O. de Andrade:** Conceptualization, Formal analysis, Investigation, Methodology, Software, Visualization. **Reza Haqshenas:** Methodology, Formal analysis, Resources. **Ki Joo Pahk:** Formal analysis, Resources. **Nader Saffari:** Supervision, Project administration.

## Declaration of Competing Interest

The authors declare that they have no known competing financial interests or personal relationships that could have appeared to influence the work reported in this paper.
